# Global Transcriptional Responses of the Toxic Cyanobacterium, *Microcystis aeruginosa*, to Nitrogen Stress, Phosphorus Stress, and Growth on Organic Matter

**DOI:** 10.1371/journal.pone.0069834

**Published:** 2013-07-23

**Authors:** Matthew J. Harke, Christopher J. Gobler

**Affiliations:** School of Marine and Atmospheric Sciences, Stony Brook University, Stony Brook, New York, United States of America; Mount Allison University, Canada

## Abstract

Whole transcriptome shotgun sequencing (RNA-seq) was used to assess the transcriptomic response of the toxic cyanobacterium *Microcystis aeruginosa* during growth with low levels of dissolved inorganic nitrogen (low N), low levels of dissolved inorganic phosphorus (low P), and in the presence of high levels of high molecular weight dissolved organic matter (HMWDOM). Under low N, one third of the genome was differentially expressed, with significant increases in transcripts observed among genes within the *nir* operon, urea transport genes (*urtBCDE*), and amino acid transporters while significant decreases in transcripts were observed in genes related to photosynthesis. There was also a significant decrease in the transcription of the microcystin synthetase gene set under low N and a significant decrease in microcystin content per *Microcystis* cell demonstrating that N supply influences cellular toxicity. Under low P, 27% of the genome was differentially expressed. The Pho regulon was induced leading to large increases in transcript levels of the alkaline phosphatase *phoX*, the Pst transport system (*pstABC*), and the *sphX* gene, and transcripts of multiple sulfate transporter were also significantly more abundant. While the transcriptional response to growth on HMWDOM was smaller (5–22% of genes differentially expressed), transcripts of multiple genes specifically associated with the transport and degradation of organic compounds were significantly more abundant within HMWDOM treatments and thus may be recruited by *Microcystis* to utilize these substrates. Collectively, these findings provide a comprehensive understanding of the nutritional physiology of this toxic, bloom-forming cyanobacterium and the role of N in controlling microcystin synthesis.

## Introduction

One of the most common bloom-forming cyanobacteria in temperate freshwater ecosystems is *Microcystis* which produces the hepatotoxin microcystin [1], [2]. Like many cyanobacteria, blooms of *Microcystis* have been associated with higher temperatures [3]–[5] and the availability of nitrogen (N) and phosphorus (P) [6], [7]. Since many freshwater ecosystems are P-limited [8]–[11], P loading is hypothesized to play a key role in the occurrence of many cyanobacteria blooms. The abundances and toxicity of *Microcystis* bloom populations have been correlated with inorganic P concentrations [5], [12] and experimental P loading has been found to significantly enhance the growth rates of toxic populations of *Microcystis* [13], [14]. Nitrogen loading may also influence the occurrence and toxicity of non-diazotrophic toxic cyanobacteria such as *Microcystis* [15], [16]. Several laboratory studies have shown that elevated N concentrations increases the growth and toxicity of *Microcystis* [15]–[17]. Recent field studies have demonstrated that N loading can promote blooms of *Microcystis* [18], [19] and laboratory experiments have shown an increase in cellular microcystin content with increasing N levels in *Microcystis* cultures [20].

While *Microcystis* blooms are associated with high levels of nutrient loading [21]–[23], concentrations of inorganic nutrients can be reduced to low levels during dense, summer blooms [24], [25] when rates of cellular uptake are at their annual maximum and rates of external loading are at a minimum [26]. Under such conditions, *Microcystis* blooms may rely on organic N and P compounds for nutrition. In aquatic ecosystems, dissolved organic nitrogen (DON) can exceed 50% of the total N pool [27] and *Microcystis* is able to grow using urea or amino acids such as alanine, leucine, and arginine as N sources [19], [28], [29]. The ability of *Microcystis* to utilize refractory forms of DON, however, is poorly understood. With regards to organic forms of P, previous research has demonstrated that cyanobacteria can assimilate and utilize organic compounds such as phosphomonoesters and phosphonates [30]–[34]. The ability of freshwater cyanobacteria to assimilate organic nutrients may give them an advantage over strictly autotrophic species during blooms when light levels or inorganic carbon levels may be greatly reduced [21].

The sequencing of the complete genome of *Microcystis aeruginosa* has yielded important insight regarding the genetic potential of this cyanobacterium [35], [36]. Transcriptome research provides a global assessment of expression patterns of all genes simultaneously and recent transcriptomic studies have provided a more detailed understanding of cyanobacterial physiology and ecology [37]–[40]. Studies applying global transcriptome profiling to some harmful algal blooms (HABs) have identified novel and important aspects of HAB ecophysiology [41]–[44]. For example, Wurch et al. [41] used sequencing of transcripts to identify the unexpected importance of purines and pyrimidines to N nutrition in *Aureococcus anophagefferens* while Straub et al. [44] revealed that more than 25% of *M. aeruginosa* genes displayed significant changes in their transcript abundance during the transition between the light and dark cycle. While investigations of how *Microcystis* responds to differing nutrient concentrations and sources at the transcript level have yet to be performed, the availability of a fully annotated genome makes such an approach tractable. The goal of this study was to obtain a comprehensive understanding of the nutritional physiology of *Microcystis.* Cultures of *Microcystis aeruginosa* (clone LE-3) were grown with low N, low P, high molecular weight dissolved organic matter (HMWDOM), and under ideal conditions while nutrients levels, cellular physiology, and gene expression patterns were assessed using whole transcriptome shotgun sequencing (RNA-seq).

## Methods

### Experimental design

Experiments were performed with *Microcystis aeruginosa* clone LE-3 (Lake Erie, USA) [45]. Treating cultures with d-cycloserine (Research Products International Corp., final concentration of 10 µM) as described in Harke *et al* [34] minimized non-*Microcystis* bacteria in cultures. Cultures were maintained in modified BG-11 medium (100 µM N, 5 µM P) illuminated by a bank of fluorescent lights that provided a light intensity of ∼100 µmol quanta m^−2^ s^−1^ on a 14∶10 light/dark cycle at 21°C.

Transcriptomic profiling experiments were performed to assess the full transcriptional response of *M. aeruginosa* LE-3 grown under low N and P, and on HMWDOM. Experimental treatments included a control in which triplicate cultures were grown with replete amounts of N and P (1.07×10^−1^ M nitrate, 1.26×10^−3^ M orthophosphate (P_i_)) and five treatments including low N (75 µM nitrate), low P (0 µM P_i_), HMWDOM (100 µM DON, 0.8 µM DOP; see below for isolation method) as the sole N and P source, a treatment with HMWDOM supplemented with 1.26×10^−3^ M P_i_ (HMWDOM+P), and a treatment with HMWDOM supplemented with 8.0×10^−5^ M ammonium (HMWDOM+N). The low N and low P treatments were used to explore transcriptional responses to N and P stress and thereby identify genes responding solely to low N (increased transcripts in the low N treatment only), genes responding to solely low P (increased transcripts in the low P treatment only), and those that responded as a consequence of a reduced growth (increased transcripts in both the low N and low P). The HMWDOM treatment was used to explore transcriptional responses when cultures were grown exclusively on organic substrates. The HMWDOM treatments supplemented with either N or P were used to assess what genes might be transcribed by *M. aeruginosa* to acquire organic forms of P or N, respectively, from HMWDOM.

HMWDOM was isolated via tangential flow filtration (TFF) [46]. TFF concentrates HMWDOM while leaving inorganic nutrient concentrations unchanged [46]. Briefly, 40 L of water were collected from Lake Agawam, NY, USA, a hypereutrophic lake that experiences dense *Microcystis* blooms [18], with permission from the Southampton Town Trustees. Lake water was sequentially filtered through 3 µm and 0.2 µm Pall Pleated Versapor® Capsule filters to remove all particles. Filtered water was then passed through a Millipore Prep/Scale Spiral-Wound Ultrafiltration module (TFF-2, 1kDa) at 25 psi until the HMWDOM was concentrated 100-fold. Isolated HMWDOM was stored at −20°C until use in experiments.

Triplicate cultures were inoculated with 3×10^5^ cells mL^−1^ and monitored for cell densities, *in vivo* chlorophyll *a* fluorescence, photosynthetic efficiency, alkaline phosphatase activity (APA), and dissolved nutrient concentrations (see below for methods) at the same time daily to avoid diel changes in gene expression and cell physiology [44]. Control cultures were harvested in exponential growth phase. For the low N and low P treatments, cultures were harvested when growth rates were depressed relative to the control treatment and photosynthetic efficiency began to decline (suggesting N limitation in the low N cultures) or when APA was high relative to the control treatment (suggesting P limitation in the low P cultures). With regards to the HMWDOM treatments, cultures were harvested when growth rates were maximal but inorganic nutrient levels were low (P_i_, nitrate, and ammonium <0.6, 3.6, and 0.8 µM, respectively), suggesting cultures were utilizing organic compounds for growth. At the time of harvest, 50 mL aliquots of each replicate in each treatment were centrifuged for 10 min. at 2,500×g at 21°C. The supernatant was poured off and resulting cell pellet was resuspended with 1 mL of remaining media and placed into a 2 mL microcentrifuge tube. The concentrated sample was centrifuged again for 10 min. at 2,500×g at 21°C and immediately flash frozen in liquid nitrogen and stored at −80°C. The entire harvest process took <30 minutes per experimental flask.

### RNA isolation and transcriptomic analyses

Total nucleic acids were extracted using the CTAB technique [47]. Extracted nucleic acids were then re-suspended in 20 µL of LoTE (3 mM Tris-HCl (pH 8.0), 0.2 mM EDTA pH8.0)) and genomic DNA was digested using an Ambion Turbo DNA-*free*™ kit according to the manufacturer’s instructions. The concentration and quality of total RNA was assessed with an Agilent Bioanalyzer™. Ribosomal RNA was removed from total RNA (∼2µg) using a Ribo-Zero™ rRNA Removal Kit for Gram-Negative Bacteria (Epicentre®) according to the manufacturer’s instructions and samples were then again reassessed with a Bioanalyzer to verify rRNA removal. Enrichment of mRNA was conducted using a TruSeq™ RNA Sample Preparation Kit v2 (Illumina®) according to the manufacturer’s instructions. Sequencing was performed by the JP Sulzberger Columbia Genome Center (New York, NY) with an Illumina HiSeq 2000 System and ∼ 8 million, 100bp, single end reads per replicate sample were mapped to a reference genome *Microcystis aeruginosa* NIES-843 [35] using TopHat [48], allowing for two mismatches per read. The relative abundance of genes and splice isoforms was estimated using Cufflinks [49] with the compatible-hits normalization option and differentially expressed genes were assessed using Cuffdiff. Cuffdiff compares FPKM (Fragments per Kilobase of exon per Million fragments mapped) values between treatments and the control and uses a beta negative binomial to model the variance across replicates allowing fold changes in expression for each gene and statistical significance (cutoff = *p*≤0.05) of these changes to be assessed [50], [51]. Transcripts which mapped to 5S, 16S, or 23S rRNA genes were removed prior to FPKM estimation, normalization, and differential expression analyses [52], [53]. Differentially expressed genes were assigned functions based upon categories used in CyanoBase (bacteria.kazusa.or.jp/cyanobase/). The blastp suite (http://blast.ncbi.nlm.nih.gov) was used to elucidate putative functions of hypothetical genes. The Illumina sequences reported in this paper have been deposited in the National Center for Biotechnology Information’s Sequence Read Archive (accession no. SRP021202).

### Culture analyses

Lugol’s iodine preserved cells were enumerated using a Beckman Coulter Multisizer™ 3 Coulter Counter® with a 50 µm aperture which allowed cell densities to be quantified with a relative standard deviation of 3%. Cell densities of selected samples were verified microscopically with a hemacytometer. Growth rates were calculated for each day of the experiment based upon changes in cell abundance according to the equation µ = Ln(N_2_/N_1_)/(t_2_–t_1_) where N_1_ and N_2_ equal the biomass at time 1 (t_1_) and time 2 (t_2_) respectively [54]. Nitrate was analyzed by reducing the nitrate to nitrite using spongy cadmium as per Jones [55]. Ammonium and phosphate were analyzed using techniques modified from Parsons *et al* [56]. Total dissolved N and P were analyzed using persulfate digestion techniques from Valderrama [57]. Urea was analyzed according to Price and Harrison [58]. These nutrient analyses provided 100±10% recovery of standard reference material (SPEX CertiPrep™) for nitrate, ammonium, phosphate, total dissolved N, and total dissolved P. Whole water samples were analyzed for the hepatoxin microcystin by first freezing samples at −80°C for 24 h and then lysing the cells using an Abraxis QuikLyse™ Cell Lysis kit for Microcystins/Nodularins ELISA Microtiter Plate according to the manufacturer’s instructions. Lysed samples were then analyzed with a colorimetric immunoassay using an Abraxis Microcystins/Nodularins (ADDA) ELISA Kit according to the manufacturer’s instructions [59]. This method provided an analytical precision of ±2% and a 96±2% recovery of spiked samples. Bulk alkaline phosphatase activity was measured for each replicate experimental sample on a Turner Designs TD-700 fluorometer (EM filter of 410–600 nm and EX filter of 300–400 nm) using 4-Methylumbelliferone phosphate (250-µM concentration) as the substrate [60]. Alkaline phosphatase activity measured by this assay has been shown to be significantly correlated with the expression of the gene encoding for alkaline phosphatase (*phoX*) in *Microcystis aeruginosa* LE-3 (*p*<0.005) [34] and provided an analytical precision of ±4%. Maximum quantum efficiency of photosystem II (PSII) was estimated from *in vivo* (F_i_) and DCMU (3,4-dichlorophenyl-1,1-dimethylurea)-enhanced *in vivo* fluorescence (F_m_) of each replicate experimental sample on a Turner Designs TD-700 fluorometer (EM filter of >665 nm and EX filter of 340–500 nm). All readings were blank corrected using BG-11 media. DCMU blocks electron transfer between PSII and PSI and yields maximal fluorescence and previous studies have demonstrated that F_v_/F_m_ can be a sensitive diagnostic of nutrient limitation, reaching a maximal value of ∼ 0.65 under nutrient replete conditions, and decreasing to less than half of that under nutrient limitation [61].

### Statistical analysis

One way analysis of variance (ANOVA) and regressions were performed using SigmaPlot version 11.0 (build 11.1.0.102). Post hoc multiple comparisons were performed with Tukey Tests and data sets which did not meet the assumption of normality or heterogeneity of variance were log transformed prior to analyses.

## Results

### Differential growth among treatments

**Figure 1:**
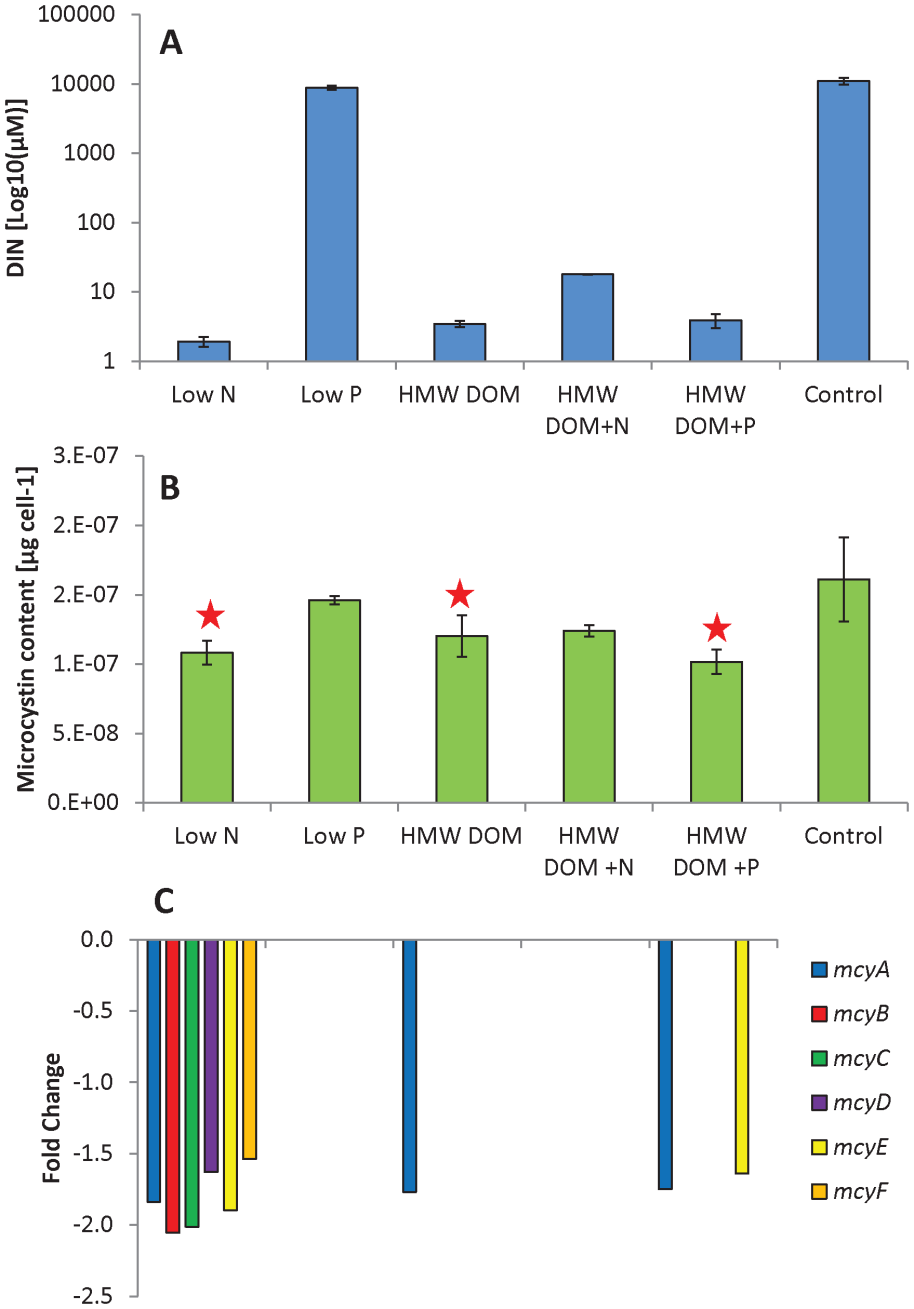
Dissolved inorganic nitrogen, microcystin levels, and *mcy* gene expression. A) Dissolved inorganic nitrogen (DIN) concentrations at the time of harvest, B) microcystin content per cell at the time of harvest and C) the fold change in gene expression relative to the control of microcystin synthetase genes. Bars represent means and error bars represent standard deviation among three biological replicates. Stars indicate significant differences relative to the control (*p*<0.05, Tukey Test).

The control cultures and cultures grown on various treatments of HMWDOM displayed similar growth rates at the time of harvest (0.44±0.06) whereas those grown on low N and low P were significantly lower (0.08±0.03 and 0.13±0.04, respectively; *p*<0.05, Tukey Test; Table 1, Figure S1). Photosynthetic efficiency of photosystem II was highest in the control (0.60±0.00; Table 1) but significantly lower and similar among other treatments at the time of harvest (0.46±0.01; *p*<0.05, Tukey Test; Table 1). Alkaline phosphatase activity was significantly higher in cultures grown without P (0.36±0.07 nmol mL^−1^ hr^−1^) compared to the control (*p*<0.005, Tukey Test; Table 1). Cultures grown on HMWDOM and HMWDOM+N displayed lower rates of APA (0.08±0.01, 0.10±0.01 nmol mL^−1^ hr^−1^, respectively; Table 1) than the low P cultures, and the HMWDOM+N APA was significantly higher than the HMWDOM+P cultures (∼0.02 nmol mL^−1^ hr^−1^; *p*<0.05, Tukey Test; Table 1). The microcystin levels in cultures grown on low N, HMWDOM, and HMWDOM+P were significantly lower than the control treatment (*p*<0.05, Tukey Test; Figure 1B) and significantly correlated with concentrations of dissolved inorganic nitrogen (DIN = nitrate+nitrite+ammonium) across all cultures (p<0.001; Figure 1 A&B).

**Table 1:** Growth rates, photosynthetic efficiency (Fv/Fm), alkaline phosphatase activity (APA) and nutrient concentrations at the time of culture harvest for each treatment.

	Low N	Low P	HMWDOM	HMWDOM+N	HMWDOM+P	Control
Growth Rate per day	0.08 (0.03)	0.13 (0.04)	0.44 (0.11)	0.51 (0.07)	0.45 (0.02)	0.37 (0.04)
Fv/Fm	0.46 (0.05)	0.48 (0.07)	0.46 (0.03)	0.44 (0.02)	0.47 (0.01)	0.60 (0.00)
APA [nmol mL^−1^ hr^−1^]	NA	0.36 (0.07)	0.08 (0.01)	0.10 (0.01)	0.04 (0.01)	0.05 (0.01)
Ammonium [µM]	1.67 (0.27)	1.56 (0.28)	0.79 (0.40)	14.0 (0.54)	0.69 (0.18)	4.91 (3.78)
Nitrate & Nitrite [µM]	0.34 (0.23)	8,799 (459)	2.66 (0.08)	3.43 (0.59)	3.55 (0.80)	10,993 (1,268)
Urea [µM]	1.02 (0.34)	2.04 (0.15)	0.54 (0.13)	0.38 (0.11)	1.32 (0.44)	1.85 (0.42)
Orthophosphate [µM]	97.7 (6.99)	1.04 (0.24)	0.25 (0.13)	0.60 (0.61)	169 (7.54)	155 (15.4)
DON [µM]	8.60 (0.95)	NA	39.6 (3.41)	48.5 (9.06)	37.0 (1.86)	NA
DOP [µM]	52.8 (14.0)	0.46 (0.26)	9.56 (0.39)	9.88 (1.04)	20.0 (9.90)	94.8 (22.1)

Values in parenthesis indicate the standard deviation among three biological replicates. NA indicates values which were not measured.

At the time of harvest, the control treatment had high levels of N and P (Table 1). In all HMWDOM treatments, DON concentrations were drawn down from 100 µM to an average of 42±6.0 µM at the time of harvest while nitrate/nitrite levels remained low, averaging 3.2±0.48 µM (Table 1). Concentrations of ammonium in these treatments were submicromolar except in the HMWDOM+N treatment in which the initial concentration of 80 µM ammonium was reduced to 14±0.54 µM at harvest (Table 1). Dissolved organic phosphorus (DOP) concentrations averaged 9.7±0.23 µM in the HMWDOM and HMWDOM+N treatments and were somewhat elevated in the HMWDOM+P (20±9.9 µM) while inorganic P was at submicromolar concentrations except in the treatments amended with P_i_ (HMWDOM+P = 170±7.5 µM P, Table 1). Nitrate/nitrite in the low N culture was drawn down to submicromolar concentrations at the time of harvest while ammonium and DON were <10 µM (Table 1). In the low P treatment, total phosphorus concentrations were <2 µM and DOP was at submicromolar concentrations (Table 1). Urea concentrations averaged 1.2±0.68 µM across treatments (Table 1).

### Transcriptomic sequencing

**Figure 2:**
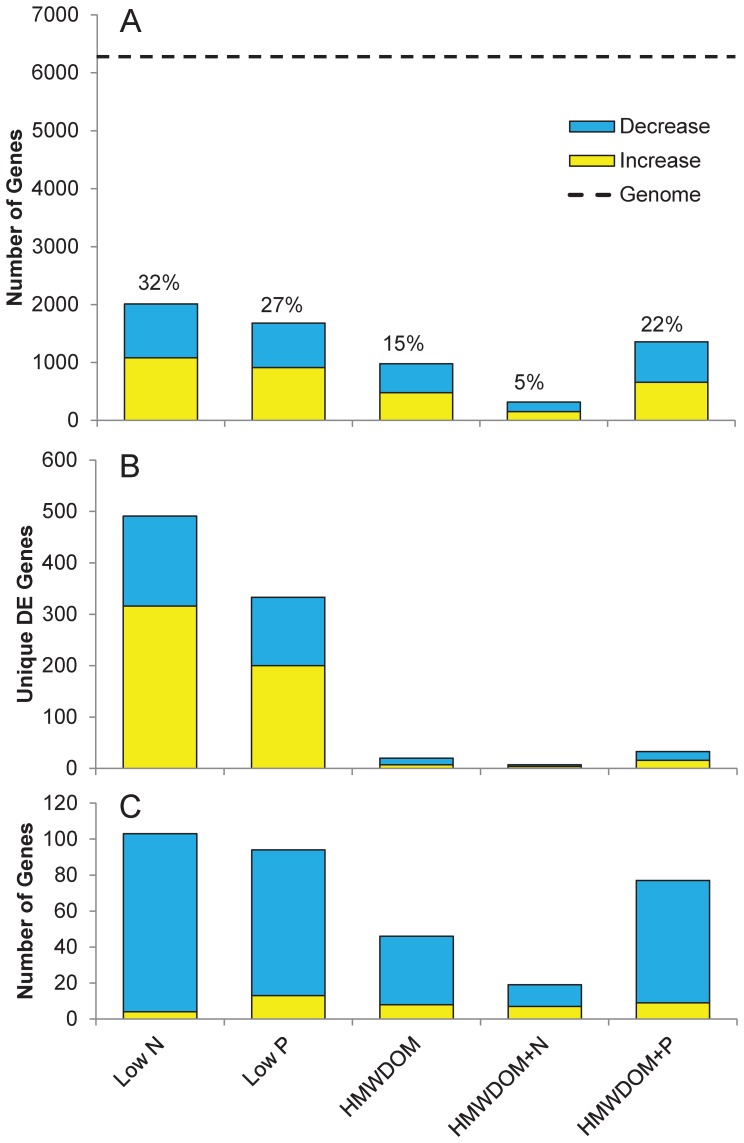
Gene expression results. A) Number of differentially expressed genes for each treatment as compared to the number of protein encoding genes in the *Microcystis aeruginosa* NIES-843 genome (dotted line) with the percent of the genome that was differentially expressed appearing above the bars. B) The number of differentially expressed (DE) genes that were unique to each treatment and C) the number of genes differentially expressed within the photosystem and respiration functional category within each treatment. Increases in transcript abundance are in yellow whereas decreases in transcript abundance are in blue. All differentially expressed genes were significant to *p*≤0.05.

Transcriptomic sequencing yielded ∼16 million, 100 base pair reads per sample. Of these, ∼8 million reads per sample mapped to 65% of genes within the reference genome *M. aeruginosa* NIES-843 [35] using a cut-off of two mismatches per read (Table S1) resulting in a coverage depth of 143X. Of the total putative protein-encoding genes in the genome (6,312), 5–32% were differentially expressed (*p*≤0.05), depending on the treatment (Figure 2A) and the majority (∼67%) of these differentially expressed genes displayed highly significant changes in transcript abundances (*p*≤0.001; Table S2). Of these highly significant genes, ∼48% had absolute fold change values ≥2 (Table S2).

### Genes responding to low N

When cultures of *M. aeruginosa* LE-3 were grown with low N, one-third of its protein encoding genes were differentially expressed (2,010 out of 6,312 genes) with 1,080 genes having significant increases in transcript abundance relative to the control and 930 genes having significant decreases in transcript abundance relative to the control (*p*≤0.05; Figure 2A). Many of the genes which had highly significant and large changes in transcript abundance (*p*≤0.001, absolute fold change ≥4; 264 genes) were involved in photosynthesis and respiration (20%) as well as hypothetical functions (28%; Table S3). Genes displaying higher transcript abundances included the global nitrogen regulatory gene, *ntcA,* (1.5-fold increase; Table 2, Figure S2) and a number of genes involved in nitrate/nitrite transport. For example, transcripts of the *nrtA, nrtB,* and *nrtC* genes encoding nitrate/nitrite transport and binding proteins had fold change values of 4.1, 5.8, and 2.3, respectively (Table 2, Figure S2). In addition, transcripts encoding nitrate reductase (*nirA*) and nitrite reductase (*narB*) were 8.9-fold and 7.8-fold more abundant under low N conditions (Table 2, Figure S2). All urea transporters genes (*urtABCDE*) had significant increases in transcripts with the exception of the *urtA* gene (Table 2, Figure S2). Furthermore, three amino-acid transporter genes (MAE 32020, MAE 26840, and MAE 26850) increased in transcript abundance (2.6-, 2.8- and 5.0-fold) under low N while a glutamate-ammonia ligase gene (*glnA*) displayed a 6.6-fold increase in transcript abundance. In addition, 19 ABC transporters displayed significant increases in transcript abundance under the low N conditions whereas many of the P acquisition and transport genes displayed significant decreases in transcript abundance (Table S3). Finally, low N cultures displayed a significant, ∼2-fold decrease in transcript abundance for most of the microcystin synthetase genes (*mcy* cassette genes, *mcy*A–*mcy*J; Figure 1C).

**Table 2:** Genes involved in nitrogen metabolism and their differential expression under each treatment relative to the control.

MAE Number	Gene Symbol	Product	Low N	Low P	HMWDOM	HMWDOM+N	HMWDOM+P
12590	*amt*	ammonium transport protein					
40020	*amt*	ammonium transport protein		2.20			
17690	*amt1*	ammonium/methylammonium permease					
40010	*amt1*	ammonium/methylammonium permease	1.58	−2.11	3.68	3.92	3.06
29150	*cphB*	cyanophycinase					
10370	*cynS*	cyanante hydratase		−1.48			
37080	*fur*	ferric uptake regulation protein					
57540	*fur*	ferric uptake regulation protein	−1.40				
08260	*gdhA*	glutamate dehydrogenase (NADP+)	−2.65	−2.17	−2.69	−2.89	−3.45
19270	*glnA*	glutamate-ammonia ligase		2.41			
09050	*glnA*	glutamate-ammonia ligase	6.56		5.66	3.91	5.39
59130	*glnB*	nitrogen regulatory protein P-II	1.99	1.94			2.70
57460	*glnB*	nitrogen regulatory protein P-II	2.79	1.85	1.71		2.25
29110	*glsF*	ferredoxin-dependent glutamate synthase	1.74		1.60		1.68
07560	*gltB*	NADH-dependent glutamate synthase large subunit	−2.46	2.66	−2.11	−2.37	−2.53
14900	*gltD*	NADH-dependent glutamate synthase small subunit	−2.75	6.13	−2.13	−2.58	−2.25
13630	*gltS*	monocomponent sodium-dependent glutamate permease	1.44		1.51		
52690	*gltX*	glutamyl-tRNA synthetase	−1.92	−1.55			−1.61
36480	*nadB*	L-aspartate oxidase					
53960	*narB*	ferredoxin-nitrate reductase	7.75		11.23	3.38	14.91
00310	*natA*	amino acid transport ATP-binding protein					
01200	*natA*	amino acid transport ATP-binding protein		−1.74			
00300	*natC*	amino acid transport system permease protein					
02170	*natC*	amino acid transport system permease protein	−1.48				
18410	*nirA*	ferredoxin-nitrite reductase	8.86		16.23	5.64	18.30
02720	*nirA*	ferredoxin-nitrite reductase					
14800	*nrtA*	ABC transporter nitrate-binding protein	4.12		14.54	7.09	15.90
14790	*nrtB*	nitrate/nitrite transport system permease protein	5.75		17.70	8.29	23.18
14780	*nrtC*	nitrate/nitrite transport system ATP-binding protein	2.34		12.80	7.11	12.68
14770	*nrtD*	nitrate/nitrite transport system ATP-binding protein			6.25	4.56	5.38
18880	*nrtB*	ABC-transporter substrate-binding protein	5.80	−2.19	3.16	2.59	3.32
18890	*nrtC*	ABC-transporter permease protein	7.51	−1.75	3.56	3.22	4.16
18900	*nrtD*	ABC-transporter ATP-binding protein	5.32		3.13	2.77	3.62
01830	*ntcA*	global nitrogen regulatory protein Ycf28	1.50	1.51			
46810	*speA*	arginine decarboxylase		−1.57			
18840	*speB*	agmatinase	−1.62	−2.34		1.88	
45220	*ureA*	urease gamma subunit	−1.98		−1.54		−1.82
45230	*ureB*	urease beta subunit	−2.18		−1.85		−2.12
61330	*ureC*	urease subunit alpha	−1.43	1.67			
04510	*ureD*	urease accessory protein D			−1.73		−1.94
41100	*ureE*	urease accessory protein E	2.45	2.98	1.89		1.84
41820	*ureF*	urease accessory protein F	1.43	1.54			
24230	*ureG*	urease accessory protein G					
06220	*urtA*	ABC-type urea transport system substrate-binding protein	−1.50				
06210	*urtB*	urea transport system permease protein	2.20		2.94	2.35	2.81
06200	*urtC*	urea transport system permease protein	2.70		2.85	2.42	2.91
06190	*urtD*	urea transport system ATP-binding protein	3.58	1.72	3.35	2.37	3.53
06180	*urtE*	urea transport system ATP-binding protein	2.91		2.26	2.14	2.57
25850		amino acid ABC-transporter permease protein	−2.91	−3.03	−2.19		−2.54
26850		amino-acid ABC-transporter permease protein	4.98		1.92	2.26	
26840		amino-acid ABC-transporter ATP-binding protein	2.75				1.72
27820		amino acid adenylation					
32020		amino-acid ABC-transporter permease protein	2.56	2.21	1.64		1.94
55930	*pipX*	PII interaction protein X	2.09	2.76			

Positive values indicate an increase in transcript abundance. Negative values indicate a decrease in transcript abundance. All values significant at *p*≤0.05.

Beyond genes related to N metabolism and transport, 103 photosynthesis and respiration genes were differentially expressed under low N. Of these, 96% displayed decreased transcript levels relative to the control with fold change values ranging from 1.4 to 62 (Figure 2C). For example, numerous genes encoding for proteins composing photosystems I and II (*psb* and *psa* gene sets) had fold change values ranging from 1.4 to 10 (Table S4). Furthermore, the abundance of transcripts encoding numerous phycobiliprotein-related genes decreased including two genes encoding for phycocyanin alpha and beta subunits (48- and 34-fold for *cpcA1* and *cpcB1*, respectively; Table S4) as well as the gene *cpcC1*, which encodes for a phycobilisome rod linker polypeptide (29-fold; Table S4). Five additional phycobiliprotein-related genes (the *apcABCDF* set) also decreased in transcript abundance with fold change values ranging from 3.4- to 10-fold (Table S4). Moreover, a gene encoding for a phycobilisome degradation protein (*nblA*) displayed a dramatic 50-fold increase in transcript abundance (Table S4). Other photosynthesis genes with large decreases in transcript abundance included two genes encoding for the large and small subunits of ribulose biphosphate carboxylase (*rbcL* and *rbcS* respectively) and genes involved in ATP synthase, carbon dioxide concentrating, and NAD(P)H dehydrogenase subunits (Table S4). Finally, the largest fold change among photosynthesis and respiration genes was that of the *petJ* gene which encodes for the cytochrome c553 protein (62-fold decrease in transcript abundance; Table S4).

Other genes related to N metabolism that displayed large changes in transcript abundance under low N included the *gifA* gene encoding for a glutamine synthetase inactivating factor which decreased transcript levels by 41-fold under low N conditions. A gene encoding for a sulfate permease protein (MAE 62080) decreased transcript levels by 15-fold (Table S3) under low N. Lastly, there were 16 genes with hypothetical functions and 7 genes of unknown function which had fold changes >10 under low N including two genes of unknown function (MAE 47790 and MAE 29200) which had 164-fold and 135-fold increases in transcript levels respectively (Table S3).

### Genes responding to low P

Under P deficiency, there were 913 genes with greater transcript abundance relative to the control and 768 genes with lower transcript abundance relative to the control (p≤0.05), representing 27% of the *Microcystis* genome (Figure 2A). Many of the genes that had highly significant and large changes in transcript abundance (p≤0.001, absolute fold change ≥4; 232 genes) were involved in translation (20%) as well as hypothetical and other functions (19 and 22% respectively; Table S5). Four genes related to P acquisition and transport (*phoX*, *sphX*, and two *pstS* genes) had large, 17–49-fold increases in transcript abundance (Table 3, Figure S3). In addition, transcript levels of numerous copies of genes within the Pst P_i_ transport system (*pstABC*) increased 1.8- to 49-fold (Table 3, Figure S3). Other genes that increased in transcript abundance under P stress included two glycogen phosphorylase genes (MAE 04590 and MAE 04570; 16- and 85-fold increase respectively; Table S5). Furthermore, a putative poly (3-hydroxyalkanoate) synthase component *phaE* gene (MAE 50040), which is involved in the production of polyhydroxybutyrate (PHB), an energy storage molecule produced by microbes in response to physiological stress, also displayed a significant increase in transcript abundance under P deficiency (12-fold change; Table S5). Finally, 5 of 7 genes encoding for sulfate binding and permease proteins increased in transcript abundance under low P (Table S5, Figure S4).

**Table 3:** Genes involved in phosphorus metabolism and their differential expression under each treatment relative to the control with positive and negative values as described in Table 2.

MAE Number	Gene Symbol	Product	Low N	Low P	HMWDOM	HMWDOM+N	HMWDOM+P
01300	*ppk*	polyphosphate kinase		3.01			
09250	*pstB*	phosphate ABC transporter ATP-binding protein					
09260	*pstA*	phosphate transport system permease protein					
09270	*pstC*	phosphate transport system permease protein	2.90	3.55	2.05		2.30
09280	*pstS*	ABC-transporter periplasmic phosphate-binding protein		3.11			
09320	*phnD*	ABC-transporter substrate-binding protein		1.43			
16640		alkaline phosphatase					
18280	*pstA*	phosphate transport system permease protein	−2.83	2.55			−1.85
18290	*pstA*	phosphate transport system permease protein	−4.02	1.83	−1.80		−2.40
18300	*pstA*	phosphate transport system permease protein	−4.53	1.79	−1.85		−2.12
18310	*pstS*	phosphate-binding periplasmic protein	−1.68	49.38	−1.66	6.15	−1.76
18340	*pstB2*	phosphate transport ATP-binding protein	−1.55	6.14			−1.61
18350	*pstB*	phosphate transport ATP-binding protein	−1.70	7.22		2.04	
18360	*pstA*	phosphate transport system permease protein	−2.33	5.07	−2.00		−1.90
18370	*pstC*	phosphate ABC transporter permease	−3.99	4.60	−2.37		−2.17
18380	*pstS*	phosphate-binding periplasmic protein	−3.40	21.81		4.09	−1.54
18390	*sphX*	phosphate transport system substrate-binding protein	−2.06	20.80		2.21	
30190	*phoX*	alkaline phosphatase	1.46	16.68		3.13	
32260	*proA*	gamma-glutamyl phosphate reductase	1.58	1.63			
32380	*pstS*	phosphate binding protein PstS homolog	1.47	2.22			1.47
43330	*phoH*	phoH like protein	−3.00	−4.09	−2.37		−2.45
47020		soluble inorganic pyrophosphatase	−4.39	−1.85			−2.02
50240		alkaline phosphatase-like protein	2.13	1.81	2.09	1.73	2.47
52210	*phnZ*	metal dependent phosphohydrolase HD region	−1.44				
52640	*sphR*	response regulator in two-component regulatory system of Pi uptake	−1.51				
52650	*sphS*	two-component sensor histidine kinase	−1.42				
52660	*phoU*	phosphate transport system regulatory protein	3.16	2.27	1.76		2.12
53740	*ppx*	exopolyphosphatase	−1.50				−1.56

All values significant at *p*≤0.05.

In a manner similar to the low N treatment, many genes involved in photosynthesis and respiration displayed decreased transcript abundance relative to the control in the low P treatment, although fold change values were, on average, 44% lower than observed in the low N treatment. In addition, some genes with lower transcript levels in the low N treatment had increased transcript levels in the low P treatment including two phycocyanin subunit genes (*cpcA2* and *cpcB2*) as well as two additional cytochrome c oxidase subunit genes (*ctaDI* and *ctaEI*). In contrast, two genes encoding for large and small subunit ribulose biphosphate carboxylase (*rbcL* and *rbcS*) had similar decreases in transcript levels to those observed in the low N treatment (9.6 and 10-fold decrease; Table S4).

There were multiple hypothetical genes that displayed large changes in transcript abundance under low P. The hypothetical gene, MAE 38890, had the largest increase in transcripts in this treatment (121-fold change; Table S5) but there were also 8 other hypothetical genes and 2 genes of unknown function that had large changes in gene expression (>10-fold increase or decrease in transcript levels; Table S5). Many of the genes (18%) with large decreases in transcripts (fold change ≥4) encoded for proteins involved in ribosomal synthesis and modification (Table S5) with the largest decrease in transcripts in the 50S ribosomal protein L10 gene (fold change of 70; Table S5).

### Genes responding to HMWDOM

**Figure 3:**
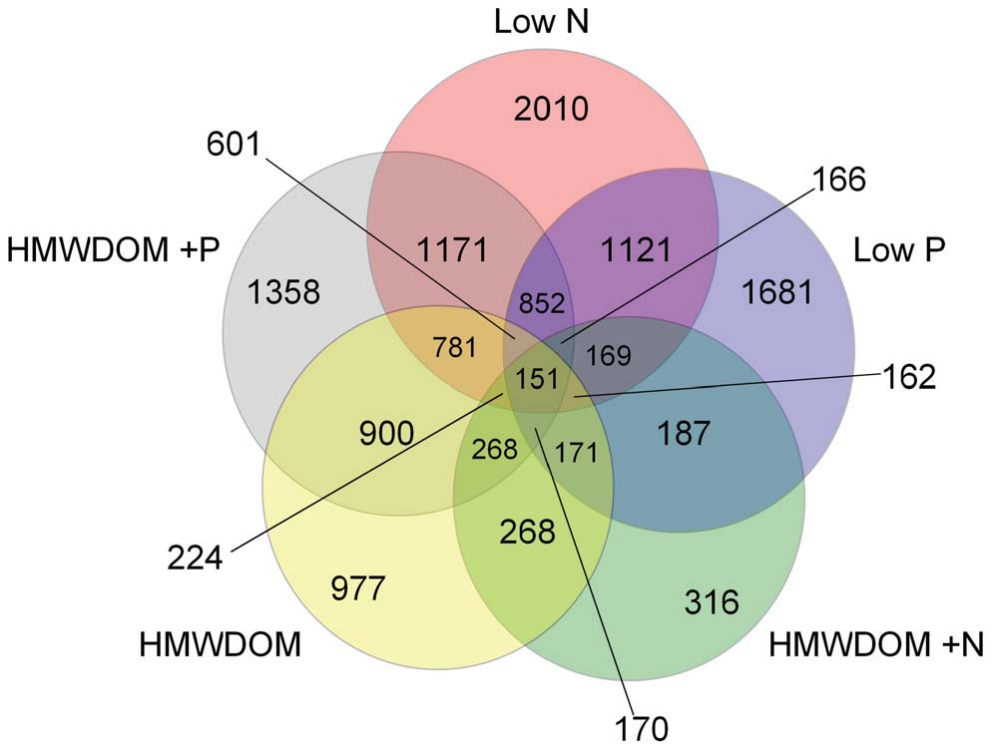
Venn diagram displaying the number of significant differentially expressed genes (*p*≤0.05) that either increased or decreased in unison in each treatment comparison represented.

When cultures of *M. aeruginosa* LE-3 were grown on HMWDOM, 964 genes were differentially expressed representing about 15% of the genome (Figure 2A), a more muted response than low N and low P treatments. When cultures grown on HMWDOM were supplemented with inorganic N, the number of differentially expressed genes decreased to 5% of the genome (316 genes; Figure 2A) whereas adding inorganic P had the opposite effect, increasing the differentially expressed gene number to 1,358 (22% of the genome; Figure 2A). In all HMWDOM treatments, genes that had highly significant and large changes in transcript abundance (*p*≤0.001, absolute fold change ≥4) largely fell within the “hypothetical” (on average 32%) and “unknown” (on average 17%) functional categories (Tables S6, S7, and S8). However, in the HMWDOM+N treatment, 27% of these highly significant and expressed genes fell into the “transport and binding proteins” functional category, whereas the percentage in the HMWDOM and HMWDOM+P treatments were smaller (13 and 8%, respectively; Table S6, S7, S8). When comparing genes in common across treatments, there were more genes with similar transcriptional patterns in the HMWDOM+P and low N treatments (1171) than any other two treatment comparisons (Figure 3). The similarity of the HMWDOM treatments to the low N treatment was also evident when comparing these treatments as the low N, HMWDOM+P, and HMWDOM had 781 genes in common whereas the low P, HMWDOM+N, and HMWDOM had only 171 genes in common (Figure 3).

Genes within the Pho Regulon, responsible for transport and assimilation of inorganic phosphate, responded dynamically to HMWDOM. In the HMWDOM and HMWDOM+P treatments, the genes *pstABC* and *phoH,* all decreased in transcript abundance while gene MAE 32380, a *pstS* phosphate binding protein, and a gene coding for the regulatory protein *phoU* (MAE 52660) increased in abundance. When cultures were grown on HMWDOM+inorganic N, the alkaline phosphatase gene, *phoX*, increased transcript levels 3.1-fold as did three inorganic phosphate binding and transport genes (two *pstS* and one *sphX* gene, 4.1-, 6.2-, and 2.2-fold increase respectively; Table 3, Figure S3).

With regards to N acquisition and transport, numerous genes increased transcript levels when grown on HMWDOM. For example, in all HMWDOM treatments, transcripts of genes involved in urea transport (*urtBCDE*) increased, on average, 2.6-fold (Table 2, Figure S2). In addition, genes involved in ammonium assimilation and transport, *glnA* and *amt1*, also had increased transcript abundances (3.9- to 5.7-fold and 3.1- to 3.9-fold respectively; Table 2, Figure S2) in all HMWDOM treatments. Lastly, genes involved in nitrate/nitrite acquisition and transport (*nrtABCD*, *narB*, and *nirA*) displayed large increases in transcript abundance when grown on HMWDOM relative to the control, with the highest fold change values observed in the cultures grown on HMWDOM and HMWDOM+P (Table 2, Figure S2).

In the HMWDOM+P treatment, there were a large number of genes differentially expressed that fell within the photosynthesis and respiration functional category. These genes displayed decreases in transcript levels that were of similar magnitude to those observed in the low N treatment (Figure 2C; Table S4). However, unlike either the low P or the low N treatments, transcript levels of five genes encoding for photosystem II D1 proteins increased, on average, 2.9-fold in all treatments grown on HMWDOM (Table S4).

The abundance of transcripts for a series of genes potentially associated with the degradation and/or transport of peptides and/or proteins significantly increased within the HMWDOM treatments. For example, the gene *hhoA*, which encodes a periplasmic protease, had higher transcript abundance in all the HMWDOM treatments but did not change in abundance in the low N or low P treatments. Transcripts encoding a tetratricopeptide TPR_2 protein (MAE 55030) increased in the HMWDOM and HMWDOM+P treatments by 25- and 17-fold, respectively (Table 4). A gene encoding for a periplasmic polyamine-binding protein (MAE 10300) displayed increases in transcript abundance in the HMWDOM and HMWDOM+P treatments that were similar to those observed in the low N treatment (Table 4). Some genes potentially associated with the degradation and/or transport of peptides and/or proteins were only differentially expressed within a single HMWDOM treatment. For example, in the HMWDOM+P treatment the caseinolytic peptidase B protein gene, *clpB1*, increased transcript levels by 1.8-fold. In the HMWDOM+N treatment the ABC transporter, MAE 51260, displayed increased transcript levels (1.8-fold), and a protease gene (MAE 19290) increased by 1.5-fold in the HMWDOM treatment (Table 4). Lastly, there were a series of uncharacterized, hypothetical genes that displayed large increases in transcript abundance relative to the control in *Microcystis* cultures exposed to HMWDOM that may be related to HMWDOM utilization (Table 4).

**Table 4:** Genes putatively involved in organic matter metabolism and their differential expression under each treatment relative to the control with positive and negative values as described in Table 2.

MAE Number	Product	Low N	Low P	HMWDOM	HMWDOM+N	HMWDOM+P
51260	ABC-transporter ATP-binding protein				1.80	
61840	ClpB protein					1.82
10300	periplasmic polyamine-binding protein	1.95	−1.73	1.58		1.64
30620	periplasmic protease			4.41	2.72	3.60
19290	protease			1.50		
55030	tetratricopeptide TPR_2	9.27	9.29	24.63		17.05
48940	two-component sensor histidine kinase			1.91	1.98	2.01
26850	amino-acid ABC-transporter permease protein	4.98		1.92		2.26
54550	ABC-transporter ATP-binding protein	1.59		1.59		1.69
14760	uncharacterized integral membrane protein			2.61	1.82	2.48
35490	hypothetical protein				2.72	
03970	hypothetical protein			4.05	2.79	4.77
15320	hypothetical protein			10.79	5.95	9.30
18150	hypothetical protein			1.72	1.83	1.76
18770	hypothetical protein			1.80	1.80	1.79
30610	hypothetical protein			21.46	9.73	16.38

All values significant at *p*≤0.05.

### Genes responding to multiple conditions

When comparing the transcriptomes of all treatments, there were 187 genes which were differentially expressed in all treatments relative to the control (Table S9) and 151 of these displayed similar expression patterns (Figure 3). For instance, *nblA*, which is involved in the degradation of phycobilisomes, had elevated transcript abundance in all conditions tested, with the highest expression of this gene in cultures grown under low N (50- fold increase, Table S9). A gene encoding for a phycobilisome core component protein (*apcF*) had lower transcript levels in all treatments with the lowest level observed in the low N treatment (9.1-fold decrease; Table S9). A putative membrane associated alkaline phosphatase (MAE 50240) had elevated transcript abundance in all treatments relative to the control with the highest fold change values observed when cultures were grown on HMWDOM or HMWDOM+P (2.1- and 2.5-fold change respectively; Table S9). A gene encoding for a CAB/ELIP/HLIP superfamily protein (MAE 08250) associated with high light, cold stress, and nutrient deprivation [62], [63] had large increases in transcript abundance (7.4–28-fold change) in all treatments (Table S9). Three genes involved in sulfate transport (MAE 31510, 31520, and 31530) had decreased transcript levels in all but the low P (which displayed a >2-fold increase; Table S9). Similarly, three other genes which may be involved in nitrate transport (MAE 18880, 18890, and 18900) displayed increased transcript levels in all treatments but the low P, averaging 4.0±1.5– fold increase (Table S9). Finally, the gene *petJ*, encoding for cytochrome c553 had large decreases in transcript abundance in all treatments with fold change values ranging from 5.5 in the HMWDOM+P treatment to 62 in the low N treatment (Table S9). When comparing transcriptional patterns between the low N and low P treatments which both displayed significantly reduced growth rates, we identified 1,121 genes with similar transcriptional patterns (Figure 3). Of these genes, 553 had decreased transcript abundance and many were involved in cellular processes such as ribosomal synthesis, photosynthesis and respiration, and amino acid biosynthesis (Table S10).

## Discussion

The role of nutrients in promoting cyanobacterial blooms has been well established [6], [22]. Numerous investigations, ranging from field studies to gene expression studies, have explored the role of nitrogen and phosphorus in promoting cyanobacterial blooms and toxicity [11], [13], [64]–[69]. By identifying the genes in *M. aeruginosa* LE-3 that are transcribed in response to N-stress, P-stress, and growth on organic matter, we have elucidated the unique molecular response of this cyanobacterium to these conditions and gained substantial insight into its nutritional physiology.

Under low N, *Microcystis* had significantly reduced growth rates and photosynthetic efficiency and a gene expression profile characteristic of N-limitation in cyanobacteria. When cyanobacteria are N-limited, 2-oxoglutarate (2-OG) accumulates intracellularly, stimulating the transcription of *ntcA* which in turn induces transcription of N assimilation genes [70], [71]. In *Anabaena* and *Synechococcus*, the *nir* operon that encodes nitrate and nitrite reductases and transporters (*nirA-nrtABCD-narB*) is expressed at high levels when ammonium is not present in the growth medium [72], [73]. This gene set also displayed high expression levels in *Microcystis* cultures which were deprived of inorganic N and this trend was most evident in cultures grown on HMWDOM where transcript levels of these genes were nearly triple that of when grown with low N, a finding consistent with the low levels of inorganic N present in these cultures. In some cyanobacteria, the presence of ammonium in the growth medium represses the expression of the *nir* operon [72], [74]. However, we still observed high levels of transcripts from this operon when there was ammonium present within the growth medium (14µM; HMWDOM+N) suggesting these genes are not repressed by elevated ammonium levels in *Microcystis* and/or that the presence of HMWDOM had a stronger effect on transcription of this operon.

Some *Microcystis* transcriptional patterns in low N cultures differed from expectations. For instance, while the annotation for *Microcystis aeruginosa* NIES-843 indicates that it has two ferredoxin-nitrite reductase genes (*nirA*; MAE 02720 and MAE 18410), only one was expressed when cultures were deprived of N (MAE 18410; 8.9-fold increase; Table 2, Figure S2). This could be due to strain differences between LE-3 and NIES-843 or that the unexpressed gene was misidentified in the original annotation. When compared to other annotated proteins, the unexpressed gene (MAE 02720) was found to be 91% similar to a gene encoding for a pecorrin-B synthase in *Microcystis aeruginosa* TAIHU98, an enzyme involved in the production of vitamin B_12_ [75], suggesting it is not a *nirA* gene. In addition, *Microcystis* may have a second set of nitrate/nitrite transport genes (*nrtBCD*; MAE 18880, 18890, and 18900) further downstream of the expressed *nirA* gene that also increased in transcript abundance under low N conditions. Assuming these genes are acting upon nitrate/nitrite, this further illustrates the complex arrangements of nitrate/nitrate assimilation genes within *Microcystis*, a finding consistent with studies of other cyanobacteria [71].

Urea is produced by the decomposition of organic compounds, is a waste product excreted by many animals, and can be found at micromolar concentrations in the natural environment [76], [77]. In all treatments but the low P, we observed significant increases in transcript abundance for genes involved in urea transport (*urtBCDE*), suggesting these genes are transcribed in response to low N conditions. Curiously, many of the urease encoding genes (*ureABCDEFG*) had decreased or unchanged transcript levels when under low N or in the presence of HMWDOM, except for two urease accessory proteins E and F which displayed significant increases in transcripts in low N, low P, HMWDOM, and HMWDOM+P. These findings are consistent with those observed in *Anabaena* in which the expression of the *urtABCDE* genes increased during N-limitation but the genes encoding urease did not [78]. In the present study, the weak response from urease may be related to the low urea concentrations in cultures (0.4–2 µM) or the possible constitutive nature of urease in *Microcystis* as has been reported for some microbes [79], [80].

Under P-stress, *Microcystis* cultures displayed elevated rates of alkaline phosphatase activity and transcripts for genes within the Pho regulon, including *pstABC*, *pstS*, *sphX*, *phoX*, and *phoU* which were all present at high levels, a finding consistent with other cyanobacteria [81] including *Microcystis* [34]. The dynamic nature of this transcriptional response was unexpected, however. For instance, multiple copies of the *pstS* and *pstABC* genes had increases in transcript levels although these increases varied between 1.8 and 49-fold. The largest fold change was observed in the *pstS* gene, MAE 18310 (high-affinity phosphate-binding protein; 49-fold change). *Microcystis*, like *Synechocystis* PCC 6803 [82], has two spatially discrete *pst* gene clusters (MAE 18340 to 18380 and MAE 09250 to 09280) but also has an additional *pstS* gene (MAE 18310) and three additional *pstA* genes (MAE 18280 to 18300) positioned downstream of one of the *pst* gene clusters [35]. When *M. aeruginosa* LE-3 was grown with low P, the *pst* gene cluster represented by MAE 18340 to 18380 had large transcript increases (4.6–21-fold change) while the second gene cluster and additional *pst* genes displayed smaller increases (1.8–3.6-fold change). This illustrates the dynamic and varied response of *Microcystis* in the face of P-stress. We originally reported that *M. aeruginosa* NIES-843 did not have the alkaline phosphatase gene *phoA* [34], however, upon further analysis, gene MAE 16640 was found to be 50% identical to *Synechococcus elongatus* PCC6301 *phoA* (E-value = 1e^−47^; accession #AAA27331). This gene, however, did not increase transcript levels under low P as did the *phoX* gene suggesting that, if it is an alkaline phosphatase, it is less active.

Under low P conditions, some cyanobacteria substitute sulfur in place of phosphorus within their membrane lipids [83]. Genes responsible for the biosynthesis of sulfolipids include members of the *sqd* gene set and in cyanobacteria, the *sqdX* gene is the most likely candidate to encode sulfolipid synthase [84]. When *Microcystis* was grown with low P, 5 of 7 sulfate transporters displayed significant increases in transcript abundance suggesting heightened requirement for sulfur within the cell. Transcripts of these transporters were significantly lower in all other treatments. There was, however, no change in *sqdX* gene transcript abundance under low P, while these transcripts decreased in abundance when cells were starved for N (Low N and HMWDOM+P treatments). Collectively, these findings may indicate that the P-deprived cells were actively sequestering sulfur but had not begun replacing their phospholipid membranes with sulfolipids [83] or that the sulfolipids synthesis gene(s) in *Microcystis* have yet to be identified.

When cyanobacteria are starved for an essential nutrient, they undergo chlorosis as photosynthetic pigments and phycobiliproteins are degraded and photosynthetic rates decline [85], [86]. In *Synechococcus* sp. strain PCC 7942, the degradation of phycobiliproteins was found to be partial under P-stress or complete under sulfur- or N-stress and the gene facilitating phycobiliprotein degradation has been identified as *nblA* [87]. Consistent with this observation, *M. aeruginosa* LE-3 displayed significantly lower photosynthetic efficiency and large transcriptional increases of the *nblA* gene when grown on low levels of inorganic N (Low N and HMWDOM+P) and significant but smaller transcriptional increases during P-limitation (low P and HMWDOM+N). Furthermore, nutrient stress generally reduces photosynthetic rates in aquatic primary producers [88], [89] and many genes relating to photosynthesis (between 12 and 99 depending on treatment) displayed decreases in transcript abundance relative to the control during nutrient stress (N or P) with both the greatest number of genes and largest decreases in transcript abundance observed in N-limited cultures and to a lesser extent in P-limited cultures. Collectively, these findings evidence the strong and broad physiological impact nutrient stress has on the photosynthetic capacity of *Microcystis*.

In the absence of inorganic forms of N and P, aquatic primary producers may rely on organic compounds for these elements. A variety of studies have explored the use of urea and amino acids by cyanobacteria [14], [90]–[94] and a few studies have explored phytoplankton growth on HMWDOM substrates. For instance, the pelagophyte, *Aureococcus anophagefferens* can grow on HMWDOM as a sole N source, depleting >25% of the available N from these compounds and displaying high rates of cell surface peptide hydrolysis [95]. Similarly, the dinoflagellate, *Alexandrium tamarense*, was able to use N from riverine HMWDOM as efficiently as nitrate [96] and iron bound to HMWDOM can contribute to the growth of some cyanobacteria [97]. No study to date, however, has explored the growth of *Microcystis* on HMWDOM substrates.

When *M. aeruginosa* LE-3 was grown on HMWDOM, there were low levels of dissolved inorganic nitrogen (DIN) and dissolved inorganic phosphorus (DIP), elevated APA, and depressed photosynthetic efficiency relative to the control suggesting the cultures were physiologically stressed by the low levels of N and P present in cultures. There was a smaller transcriptional response to HMWDOM by *Microcystis* suggesting it has fewer unique biochemical pathways for directly utilizing organic compounds compared to the wider array of pathways recruited to grow under low N and P. For instance, among genes uniquely expressed in a single treatment, there were 491 and 333 such gene transcripts present at significantly different abundances under low N or low P, respectively, whereas the numbers of unique transcripts present at significantly different levels when grown on HMWDOM, HMWDOM+N, and HMWDOM+P were smaller (20, 7, and 33, respectively; Figure 2B). This pattern was also evident in the whole transcriptomic response (Figure 2A), supporting the conclusion that *Microcystis* possesses a broad array of genes to response to nutrient stress, but recruits a significantly smaller gene set to grow on organic matter.

The transcriptional responses of *Microcystis* to the three HMWDOM treatments were, in some respects, similar to the responses of *Microcystis* grown with low N and/or low P. For example, the low N and all three HMWDOM treatments, had large transcript increases of genes within the *nir* operon, urea transport genes (*urtBCDE*) and the ammonium permease gene, *amt1*, suggesting that *Microcystis* responded to HWMDOM in a manner similar to N-stress, even when there was excess ammonium present (HMWDOM+N). When grown on HWMDOM, *Microcystis* had more genes in common with low N and HMWDOM+P than any other treatment suggesting a stronger response to N-stress than P-stress (Figure 3). With regards to P-stress, the low P and HMWDOM+N treatments both displayed the highest levels of alkaline phosphatase activity and increased transcript levels in the alkaline phosphatase *phoX* as well as the Pst P_i_ transport system (*pstABC*), and the *sphX,* phosphate transport gene, demonstrating that the addition of N stimulated P-stress in cultures grown with HWMDOM.

Some genes with higher transcript abundances in various treatments were likely important for transporting and metabolizing N or P from organic compounds. For instance, the gene MAE 55030, which encodes for a protein within the CHAT peptidase domain and is important for protein degradation [98], had higher transcript levels in the low N, HMWDOM, and HMWDOM+P treatments suggesting it may be involved in degrading HMWDOM compounds. The low N, HMWDOM, and HMWDOM+P treatments also had more transcripts of gene MAE 10300, which encodes for a periplasmic polyamine-binding protein. Polyamines are present in aquatic environments primarily as putrescine, spermidine, and spermine [99] and MAE 10300 may be important for harvesting exogenous sources of these substrates. Other genes with higher transcript levels under low N, HMWDOM, and HMWDOM+P treatments and potentially involved in DON transport included an amino-acid transporter (MAE 26850) with a 64% identity to a glutamine ABC transporter in *Synechocystis* sp. PCC 6803 (E-value = 0.0) and an ABC transporter in the C39 peptidase family (MAE 54550) with putative roles in cleaving double-glycine leader peptides from bacteriocins [100]. We also observed two genes (MAE 51260 and 35490) that displayed significantly higher transcript abundance in only the HMWDOM+N treatment suggesting they may be involved specifically in DOP utilization. Gene MAE 35490 is 99% similar to a gene in two other strains of *Microcystis* (strain PCC 9701 and T1-4, E-value = 2e^−104^ each) and has been annotated as having similarity to an ATPase in *Trichodesmium erythraeum* (strain IMS101). Gene MAE 51260 is most similar to a putative lipopolysaccharide transport protein in other strains of *Microcystis* (E-value <3e^−170^) which may be involved in transporting carbohydrates, organic alcohols, and acids [101]. Collectively, the higher transcript levels of these genes in the absence of high levels of inorganic N and/or P suggest they may assist in transporting and degrading organic compounds.

Growth rates are linked with a number of biochemical processes in phytoplankton and can be influenced by temperature, nutrient availability, and light [54], [102]. With regards to gene expression, there is evidence of growth-rate dependent regulation of certain genes within the cell such as transcription of ribosomal RNA [103] and it has been shown that the expression of proteins can also affect growth rate [104]. During this study, the low N and low P treatments were harvested when their growth rates had declined relative to the control. This allowed for identification of genes that responded as a consequence of a reduced growth (those responding to both low N and low P) compared to those genes responding solely to low N or solely to low P conditions (as discussed above). When comparing transcriptional patterns between the low N and low P treatments, 1,121 genes displayed similar transcriptional patterns (Figure 3). Approximately half of these genes experienced decreased transcript abundance and many of these were involved in ribosomal synthesis and amino acid biosynthesis suggesting these genes may be responding to reduced growth rate rather than nutrient stress.

Heterotrophic bacteria are known to inhabit the mucilage of *Microcystis* spp colonies [105] and play a key role in nutrient remineralization [106], [107]. In this study, cultures were initially treated with the antibiotic cycloserine to reduce the presence of any heterotrophic bacteria that may have been present. Subsequent epifluorescent microscopy confirmed the success of this treatment, although we cannot rule out growth of some heterotrophic bacteria within the cultures during the course of the experiment. Since our transcripts were specifically mapped to the genome of *Microcystis*, this cyanobacteria was exclusively responsible for the transcript data presented in this study.

Finally, with regards to toxin production, under low N, the microcystin content per *Microcystis* cell was significantly reduced and transcripts of the microcystin synthetase genes (*mcy* cassette) were less abundant suggesting that microcystin synthesis is dependent upon a sufficient N supply. Some of these genes also had decreased transcript levels in the HMWDOM and HMWDOM+P treatments that also had low levels of inorganic N, affirming the importance of high N levels for microcystin synthesis (Figure 1A and 1B). Microcystin is a N-rich compound (10 N atoms per molecule) and studies have found microcystin can represent up to 2% of cellular dry weight of *Microcystis* [108]. Beyond the N in the toxin, toxic *Microcystis* strains such as LE-3 will have additional N requirements associated with the enzymes involved in the synthesis of microcystin [109] as well as with additional light-harvesting pigments they may possess [110]. These observations are also consistent with previous observations that N-enrichment promotes microcystin production by *Microcystis* [111] and increases in the abundance of toxic strains of *Microcystis* [14], [112], and that cells become less toxic during N-starvation [16].

### Conclusions

In summary, whole transcriptome sequencing of *Microcystis aeruginosa* during growth with low N, low P, and HMWDOM has provided robust insight regarding the nutritional physiology of this cyanobacterium. Under low N, there was a large increase in transcript levels of genes related to N assimilation and a decrease in transcript levels in genes related to photosynthesis and microcystin synthesis. There is much diversity in the organization of the *nir* operon within freshwater cyanobacteria (as reviewed by Ohashi et al. [71]) and we have expanded upon this identifying a new and possible more active *nirA* gene and a putative second copy of the *nrtBCD* genes within *Microcystis*. Under low P, the Pho regulon was induced leading to large increases in transcript levels of the alkaline phosphatase *phoX*, the Pst P_i_ transport system (*pstABC*), and the *sphX* gene. These global analyses revealed that *Microcystis* has two *pst* gene clusters (*pstSCAB*), additional copies of the *pstA* and *pstS* genes, and a *phoA* gene that was not expressed, suggesting a broad and active, yet complex, P harvesting ability. Additionally, genes encoding for sulfate binding and permease proteins had increased transcript levels under low P, while genes involved in photosynthesis had lower transcript levels. In contrast to N and P, there was a smaller transcriptional response to growth on HMWDOM. In many cases, the transcriptional patterns of the HMWDOM with N or P treatments tended to mirror the low P and low N treatments, respectively. There were, however, transcripts of multiple genes specifically associated with the transport and degradation of organic compounds that were significantly more abundant within HMWDOM treatments that may be important for utilizing organic compounds.

The transcriptomic profile of *Microcystis aeruginosa* LE-3 grown with low N, low P, and HMWDOM is a first step toward providing a broader understanding of the nutritional physiology of this toxic, bloom-forming cyanobacterium. Future studies of this species may target a finer scale time series of its transcriptomic response or additional strains of this species. Future proteomic studies will help verify transcriptional patterns observed in this study and future ecosystem studies will be useful for assessing the extent to which the transcriptional patterns displayed by cultures are manifested within wild populations of *Microcystis* during bloom events.

## Supporting Information

Figure S1 **Daily changes in** ***Microcystis*** **cell densities during experiments.** Red boxes indicate the day cells were harvested for transcriptomic sequencing.
(TIFF)

Figure S2 **Fold change in transcripts of genes involved in nitrogen metabolism.** Bars represent the fold change in gene expression relative to the control treatment (*p*≤0.05).
(TIFF)

Figure S3 **Fold change in transcripts of genes involved in phosphorus metabolism.** Bars represent the fold change in gene expression relative to the control treatment (*p*≤0.05).
(TIFF)

Figure S4 **Fold change in transcripts of genes involved in sulfate binding and transport within the low P treatment.** Bars represent the fold change in gene expression relative to the control treatment (*p*≤0.05).
(TIFF)

Table S1 **Summary of transcriptomic sequencing results.**
(XLSX)

Table S2 **Statistical differences in gene expression.** Values represent the number of genes and percentages represent the percent of the total differentially expressed genes.
(XLSX)

Table S3 **Differential expression results for the low N treatment compared to the Control treatment.**
(XLSX)

Table S4 **Genes within the photosynthesis and respiration functional category.** Columns C through G contain fold change values relative to the control with positive and negative values as described in Table 2.
(XLSX)

Table S5 **Differential expression results for the low P treatment compared to the Control treatment.**
(XLSX)

Table S6 **Differential expression results for the HMWDOM treatment compared to the Control treatment.**
(XLSX)

Table S7 **Differential expression results for the HMWDOM+N treatment compared to the Control treatment.**
(XLSX)

Table S8 **Differential expression results for the HMWDOM+P treatment compared to the Control treatment.**
(XLSX)

Table S9 **Genes showing differential expression in all treatments.** Columns B through F contain fold change values relative to the control with positive and negative values as described in Table 2. All values significant to *p*≤0.05.
(XLSX)

Table S10 **Genes showing similar differential expression in the low N and low P treatments.** Columns B through F contain fold change values relative to the control with positive and negative values as described in Table 2.
(XLSX)
