# Supplementary figures and images for: Global Transcriptional Responses of the Toxic Cyanobacterium, Microcystis aeruginosa, to Nitrogen Stress, Phosphorus Stress, and Growth on Organic Matter
Source: PLoS One. 2013 Jul 23;8(7):e69834. doi: 10.1371/journal.pone.0069834 (PMC3720943; doi:10.1371/journal.pone.0069834)

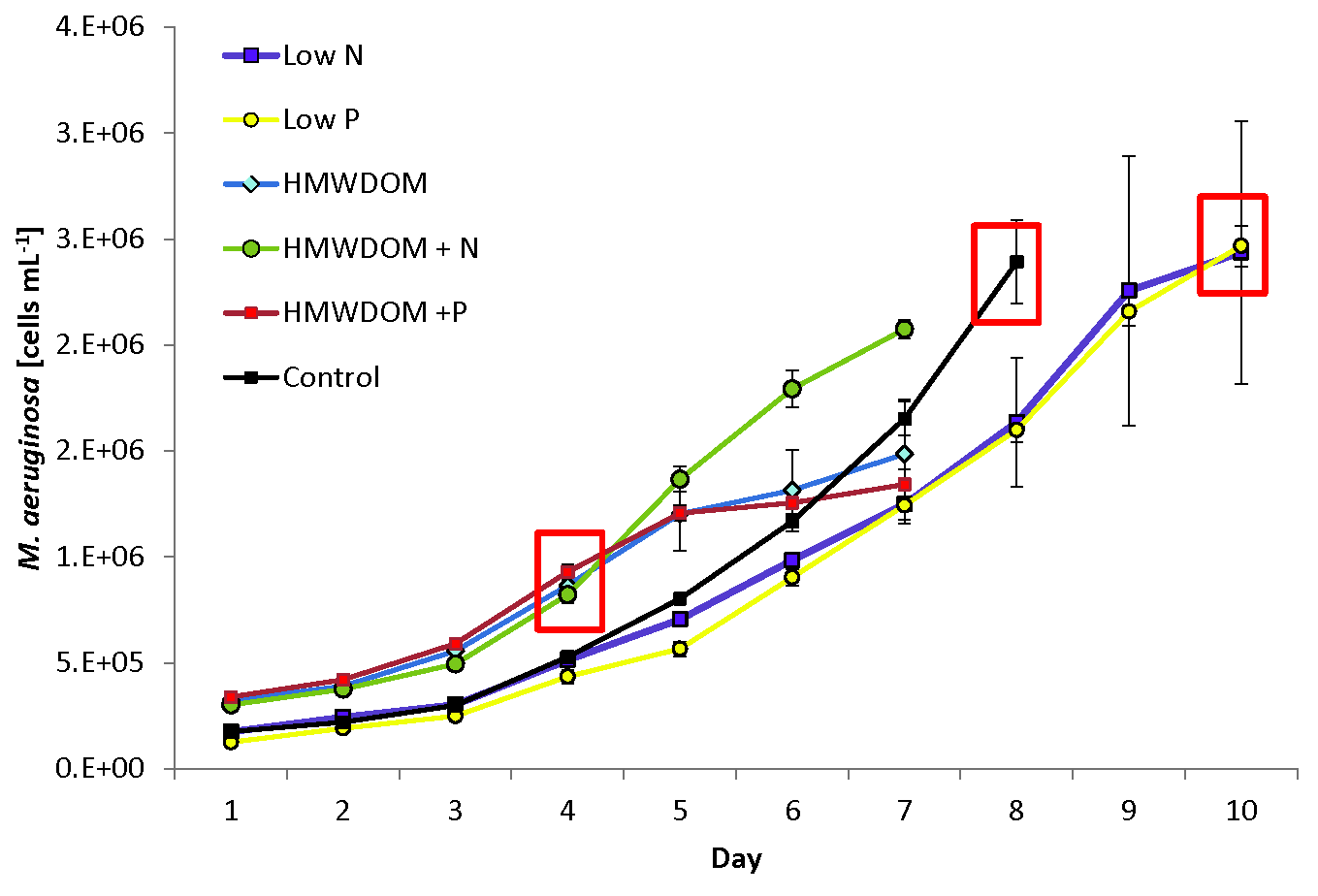

Supplement: Figure S1 — Daily changes in Microcystis cell densities during experiments. Red boxes indicate the day cells were harvested for transcriptomic sequencing. (TIFF) [file pone.0069834.s001.tiff]

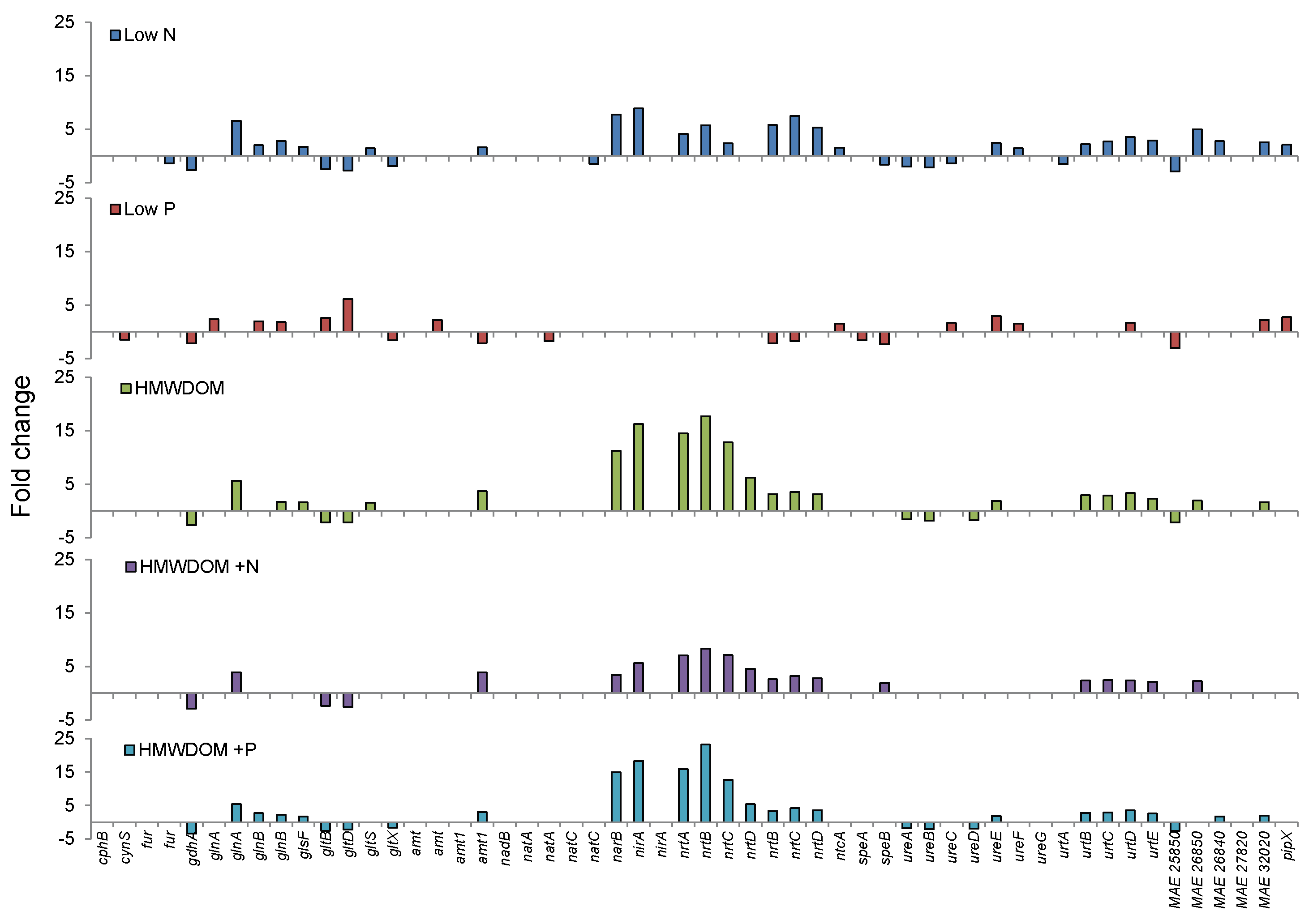

Supplement: Figure S2 — Fold change in transcripts of genes involved in nitrogen metabolism. Bars represent the fold change in gene expression relative to the control treatment (p≤0.05). (TIFF) [file pone.0069834.s002.tiff]

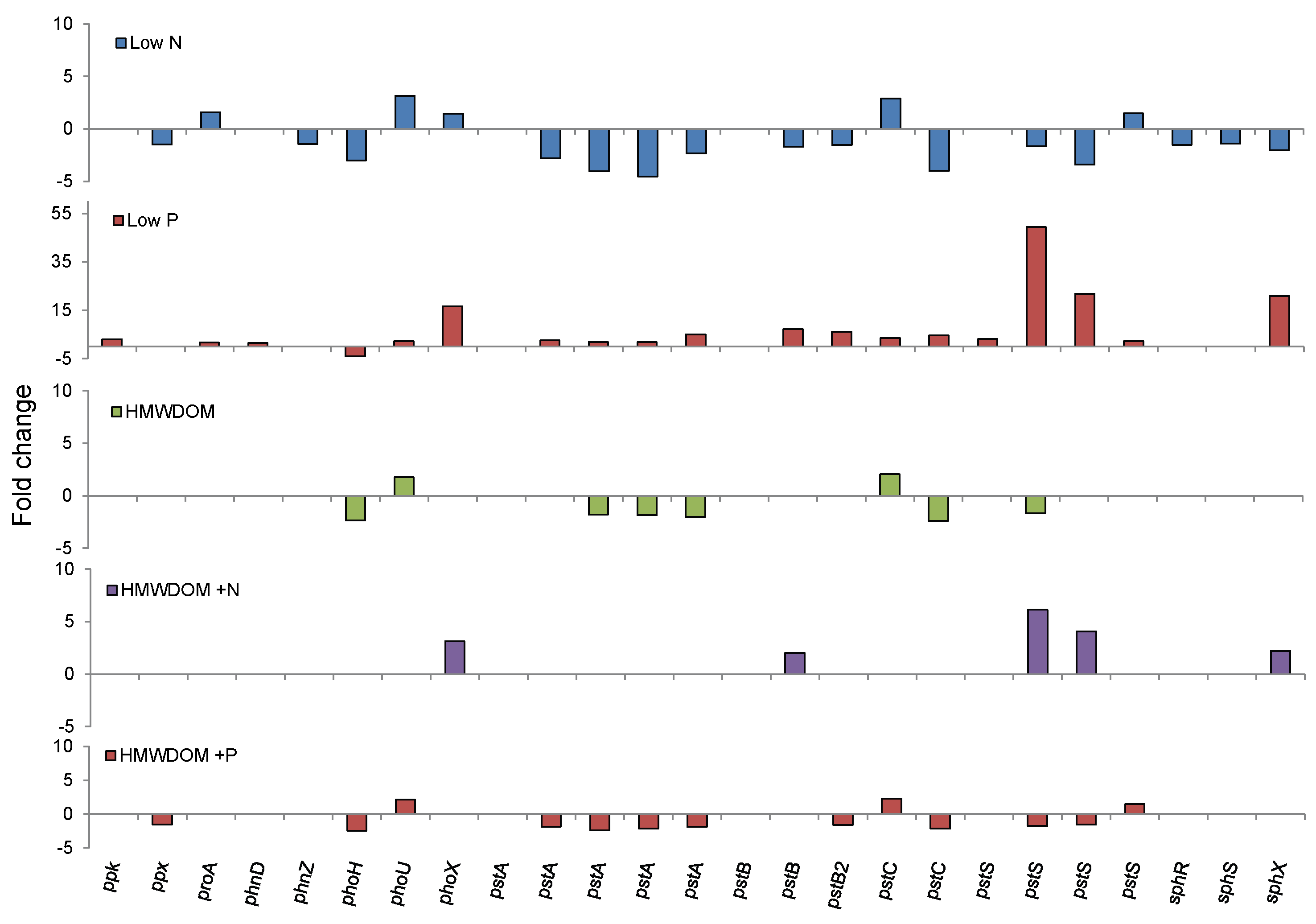

Supplement: Figure S3 — Fold change in transcripts of genes involved in phosphorus metabolism. Bars represent the fold change in gene expression relative to the control treatment (p≤0.05). (TIFF) [file pone.0069834.s003.tiff]

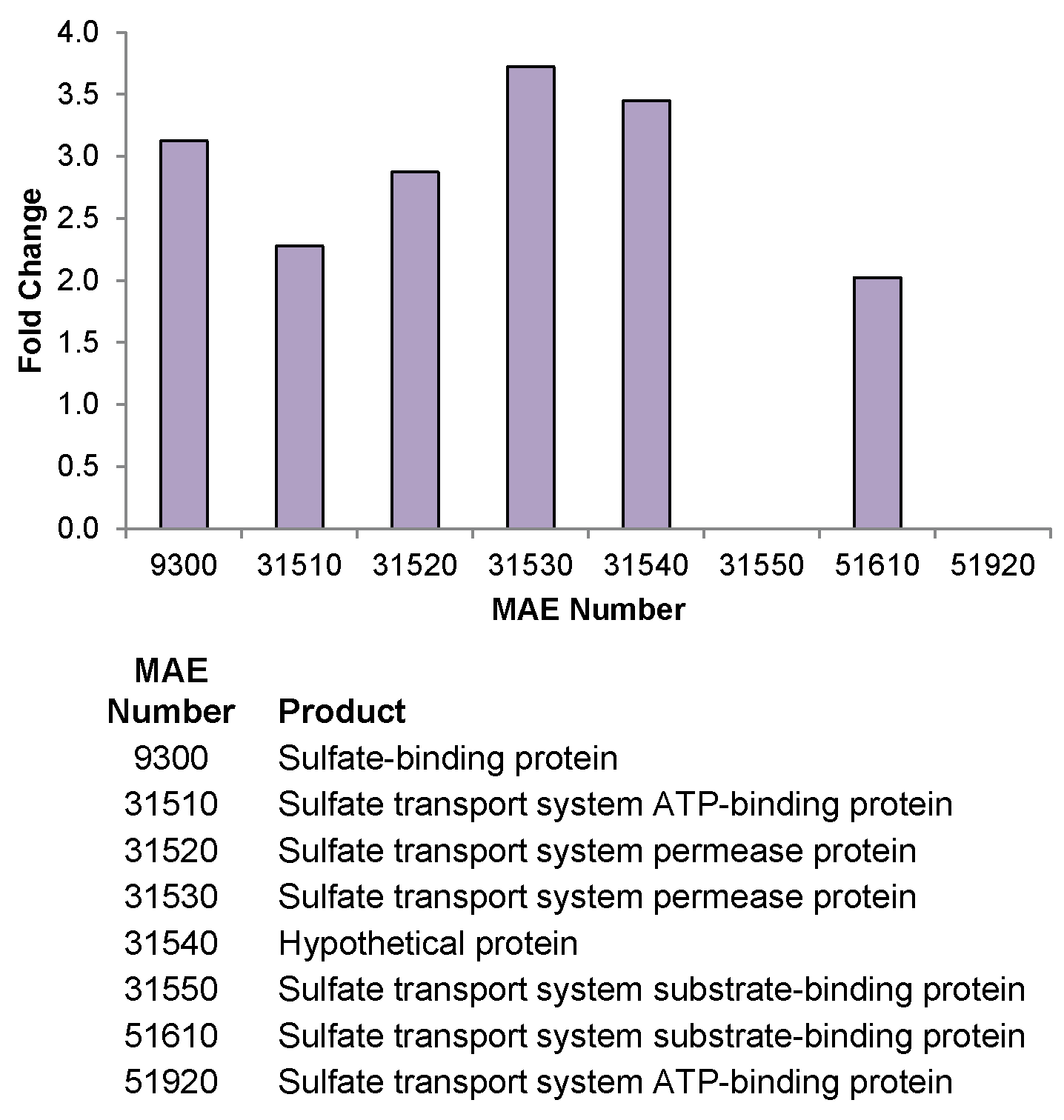

Supplement: Figure S4 — Fold change in transcripts of genes involved in sulfate binding and transport within the low P treatment. Bars represent the fold change in gene expression relative to the control treatment (p≤0.05). (TIFF) [file pone.0069834.s004.tif]
